# Level of awareness of mammography among women attending outpatient clinics in a teaching hospital in Ibadan, South-West Nigeria

**DOI:** 10.1186/1471-2458-13-40

**Published:** 2013-01-16

**Authors:** Millicent O Obajimi, Ikeoluwapo O Ajayi, Abideen O Oluwasola, Babatunde O Adedokun, Adenike T Adeniji-Sofoluwe, Olushola A Mosuro, Titilola S Akingbola, Oku S Bassey, Eric Umeh, Temitope O Soyemi, Folasade Adegoke, Idiat Ogungbade, Chinwe Ukaigwe, Olufunmilayo I Olopade

**Affiliations:** 1 Department of Radiology, University College Hospital, Ibadan, Nigeria; 2 Department of Epidemiology, Medical Statistics and Environmental Health, University College Hospital, Ibadan, Nigeria; 3 Department of Pathology, University College Hospital, Ibadan, Nigeria; 4 Department of Family Medicine, University College Hospital, Ibadan, Nigeria; 5 Department of Hematology, University College Hospital, Ibadan, Nigeria; 6 Department of Medical Oncology And University of Chicago, Chicago, Illinois, USA

**Keywords:** Mammography, Women, Awareness, Nigeria

## Abstract

**Background:**

Mammography has been used in developed countries with considerable success but very little is known about this imaging modality in low resource settings. This study examined the level of awareness of mammography and determined factors influencing the level of awareness.

**Methods:**

We conducted a hospital based cross sectional study to investigate the level of awareness of mammography among 818 randomly selected women attending the General Outpatient clinics (GOP) of the University College Hospital (UCH), Ibadan, Nigeria. Independent predictors of level of awareness of mammography were identified using multiple logistic regression analysis.

**Results:**

The proportion of women who ever heard of mammography was 5%, and they demonstrated poor knowledge of the procedure. Those with primary or secondary levels of education were about three times less likely to be aware of mammography when compared with those with tertiary level of education (OR = 0.3, 95% CI, 0.12 – 0.73). Also, participation in community breast cancer prevention activities (OR = 3.4, 95% CI, 1.39 – 8.36), and previous clinical breast examination (OR = 2.34, 95% CI, 1.10 – 4.96) independently predicted mammography awareness. Newspapers and magazines appeared to be the most important sources of information about mammography screening.

**Conclusion:**

The level of awareness of mammography is poor among women attending outpatient clinics in the studied population. Interventions promoting awareness of this screening procedure should give particular attention to the illiterate and older women while clinicians performing breast examinations should utilize the opportunity to inform women about the mammography procedure. Promotion of educational articles on breast cancer and its screening methods via media remains vital for the literate.

## Background

Breast cancer is by far the most frequent cancer of women, ranking second overall when both sexes are considered [1]. Annually many women die of the disease in Nigeria with majority of them presenting in the late stages of the disease when very little or nothing can be done to stop disease progression [2]. Mortality from breast cancer is preventable if the disease can be diagnosed early [3]. There are different methods of breast cancer screening which include breast self- examination (BSE), clinical breast examination and mammography each with its advantages and disadvantages. Mammography is the mainstay of screening for breast cancer. Although not a new screening method, it is yet to be widely available especially in the low- resource countries including Nigeria. The level of awareness and utilization of these screening methods in Nigeria, as in most other developing countries is quite poor [4]. For example the practice of breast self- examination and utilization of other screening methods has generally been poor even among nurses and other female health workers [5,6].

Mammography is the only breast screening procedure for which empirical evidence exists to have significantly reduced breast carcinoma mortality by about 63% [7]. However there are still challenges concerning its use such as costs, false positivity, pain during the procedure and risk of radiation exposure. Evidence supporting the usefulness of mammographic screening is strongest for women between 50 and 69 years of age and it has been recommended that screening should be routinely recommended for women in this age group [8].

Despite the benefits of mammography, several previous studies demonstrated poor knowledge, attitudes and utilization among variable study populations in the developing world [6,9-11]. Several factors have been identified as influencing the level of knowledge and utilization of screening services in general. Socio-demographic characteristics such as younger age group [12] and education [4,13] have been reported with higher levels of awareness and utilization of screening services commoner among the educated and those with high socioeconomic class. It has also been shown that a positive family history of breast cancer may not translate into greater worry about breast cancer or early detection priorities [14] while a significant association between family history and practice of breast self- examination was reported elsewhere [15]. Other variables associated with utilization of breast cancer screening services included access to physician care [16], perception of organizational and structural factors [17] and means of financing health.

Many of the other studies on breast cancer screening in Nigeria have focused on clinical breast examination and breast self- examination. Some conducted studies on knowledge, attitudes and practice of mammography among different professionals and social cadres in Nigeria [6,9,10]. They all agreed that women still present late due to a low level of awareness or poor knowledge about the use and benefits of mammography. Akhigbe *et al.*[6] found a high (80.7%) awareness level of mammography as a diagnostic tool but adequate knowledge of the procedure was lacking (overall mean knowledge score was 3.74 ± 2.30) and only 23.7% of the respondents had good knowledge about the importance of screening mammography for early detection of breast cancer.

In similar study among female healthcare professionals in Lagos unlike the study in Benin, female doctors were the only professional group that had a satisfactory knowledge of breast cancer risk factors with a mean knowledge score of 74% [5]. The thrust of this present study is to identify the factors that influence awareness of mammography among patients attending the outpatient department of a tertiary hospital. Understanding the relationship between patient’s characteristics, perceptions, level of utilization of health services and practices related to breast care and mammography awareness could assist in identifying key variables in planning interventions for women in Nigeria and similar low resource settings.

## Methods

### Study area and sample

A hospital based cross sectional study investigating the level of awareness of women attending the General Outpatient clinic (GOP) of the University College Hospital (UCH), Ibadan, Nigeria. The UCH is a 700 bed hospital, the largest in Nigeria which serves as a main referral center from hospitals in the south west region and other African countries. The GOP is the outpatient section dealing with all new cases excluding accidents and emergencies. Patients are sorted and referred for specialized care after consultation at the GOP. Women aged 18 years and above attending the GOP for care were studied. This study was part of a larger study of knowledge, attitudes, behaviors about breast cancer and utilization of breast cancer screening services. Sample size for estimating single proportions was used to determine the number of women studied which was based on the proportion of women who could correctly identify breast self- examination as a method of detection of breast cancer of 43.2%. Assuming a precision of 5% and a type 1 error rate of 5% for a 2 sided test the original study recruited 818 women which the present study used. The systematic random sampling method was used to select those interviewed.

### Data collection and ethical approval

Interviewer administered semi- structured questionnaires were used to obtain information from the women about their socio-demographic characteristics as well as their knowledge and utilization of breast cancer screening services. The questionnaire was organized into the following sections: Bio-data; Breastfeeding history; Contraceptive use; Knowledge and cost of cancer screening; General health prevention practices; Breast cancer knowledge; Practices related to screening. The questionnaire was pretested among 30 women presenting at the Medical Outpatient clinic of the UCH. The main outcome variable in this study is mammography awareness. The women were asked the question ‘Have you ever heard of mammography?’ For women with no formal education, the most accurate description of the procedure in the local language was given based on a consensus determined by the authors. Those who had ever heard of mammography were then asked what they knew about it, how often it should be done, the risks associated and if they will be willing to have a mammography done. Ethical approval was obtained from the University of Ibadan/University College Hospital Ethics Committee, Ibadan.

### Data management analysis

The questionnaires were edited after each interview and were identified using identification numbers in place of participant’s name to ensure participant’s confidentiality. Data was entered using SPSS version 16, checked for errors and necessary editing carried out. Means, frequencies and proportions were used to summarize variables. With awareness of mammography as the dependent variable associations between categorical predictors were tested using the chi square tests. The variables significant at 5% on chi square tests were then entered into a logistic regression analysis to identify independent predictors of awareness. Level of significance for all tests was at 0.05 (95% confidence interval).

## Results

There were 818 women studied and majority (84%) was of the Yoruba tribe. The mean age of the women was 40.2 years (SD = 14.6) with a range of 18 – 86 years. The age distribution revealed that about 27% were aged less than 30 years followed by 24% in the age group 30 to 39 years, 20% and 16% in the age group 40–49 years and 50–59 years respectively while the remaining12.7% constituted women aged 60 years and above. About 80% of the women had some form of formal education with 31.8% having attained tertiary level of education. About 64.8% were currently in marital unions while 21.8% of the women had never married and 13.4% were either widowers or divorcees. There were 227 women (27.8%) who had never had children while 47.1% had 1–4 children and 25.2% had 5 or more children.

Forty two (5.1%) women had previously heard of mammography. A greater proportion, 30 (71.4%) of these 42 women had tertiary level of education. Two women knew that mammography was a picture or photograph of the breast while only one said that it is an ‘X ray taken to check the condition of the breast’. There were six who knew that it is used for breast cancer detection while the remainder (33, 78.6% of those aware) only heard about it as a screening method for breast disease but were unaware of what it is or how it is done. The nine women with any knowledge of mammography were all less than 40 years of age and had attained tertiary level of education. Among the 42 respondents who ever heard of mammography, 16 (38%) indicated willingness to have a mammogram while 6 (14.3%) knew there is a risk in having mammography. None of the women had ever had a mammogram.

The proportions of women who had ever heard of mammography for categories of selected socio-demographic characteristics are shown in Table 1. There was a higher level of mammography awareness among those less than 40 years of age compared to older women (p = 0.021), those with tertiary level of education compared to lower educational levels (p < 0.001) and lower proportion among those with five or more children compared with women with lesser number of children (p = 0.006). There were no significant associations between awareness and ethnicity, marital status or religion (Table 1).

**Table 1:** Relationship between awareness of mammography and socio-demographic characteristics

**Variable**	**Aware of mammography n (%)**	**Total**	**P value**
Age (years)			
Less than 30	16(7.2)	222	0.021
30-39	16(8.0)	200	
40-49	5(3.0)	164	
50-59	4(3.1)	128	
60+	1(1.0)	104	
Level of education			<0.001
None	3(1.8)	167	
Primary	3(2.0)	149	
Secondary	6(2.5)	242	
Tertiary	30(11.5)	260	
Parity			0.006
None	16(7.0)	227	
1-2	9(6.5)	138	
3-4	16(6.5)	247	
5 or more	1(0.5)	206	
Ethnicity			
Yoruba	38(5.5)	687	0.239
Others	4(3.1)	131	
Marital status			
Single	15(8.4)	178	0.078
Married	22(4.2)	530	
Widowed/divorced	5(4.5)	110	
Religion			
Islam	15(4.5)	333	0.437
Christian	27(5.7)	470	

There was also a significant relationship between having heard or seen anything recently about breast cancer and mammography awareness (p < 0.001). Level of awareness significantly differed by source of information about breast cancer. The study showed the highest proportion of awareness of mammography among those who obtained information from newspapers or magazines followed by friends or colleagues, hospital, and radio or television. There was a significantly higher proportion of awareness among those who ever had discussions about breast cancer compared to those who had not (p < 0.001). Those with a family history of cancer had higher levels of awareness but this was not statistically significant. The number of women who took part in a breast cancer detection program was small. No significant association was found between mammography awareness and participation in a breast cancer program (Table 2).

**Table 2:** Relationship between awareness of mammography and other selected characteristics

**Variable**	**Aware of mammography n (%)**	**Total**	**P value**
Family history of cancer?			0.434
Yes	4(7.4)	54	
No	38(5.0)	764	
Source of information about breast cancer			
Newspaper/magazine	7(10.6)	66	0.003
Radio/television	6(2.8)	212	
Friends/Colleagues	9(8.8)	102	
Hospital	4(4.2)	95	
Others	13(8.7)	150	
Never heard about cancer recently	3(1.6)	193	
Took part in a breast cancer detection programme?			
Yes	2(12.5)	16	0.178
No	40(5.0)	802	
Ever had breast cancer discussions with other women?			
Yes	20(10.6)	188	<0.001
No	22(3.5)	630	
Know one or more breast cancer symptoms?			
Yes	28(8.5)	331	<0.001
No	14(2.9)	487	
Know one or more risk factors for breast cancer?			
Yes	1(0.4)	284	<0.001
No	41(8.0)	511	

Composite variables for knowledge of any of the symptoms of breast cancer and those with correct responses to risk factors for breast cancer were derived from the question items on knowledge and the associations of the variables with awareness of mammography are also shown in Table 2. There was significantly higher level of awareness of mammography among those with higher knowledge of breast cancer risk factors or symptoms.

The associations between level of awareness and selected variables are shown in Table 3. Women who perceived themselves as likely to have breast cancer had a significantly higher level of awareness compared to those who felt they are not likely to have the disease (p < 0.001). About 18% of those who had heard or seen anything about cancer prevention in the community compared to 4% of those who had not had such exposure reported mammography awareness, a difference which was statistically significant (p < 0.001). There was also a significant association between having had a previous breast examination and mammography awareness. In particular about 7% of women who had practiced breast self- examination were aware of mammography compared to about 3% of those who do not practice BSE (p = 0.019). Similarly women whose breasts had been previously examined by a doctor or a nurse for lump had higher level of mammography awareness compared to those whose breasts had not been previously examined (p < 0.001). There was a significant association between awareness of breast ultrasonography and mammography awareness. Even though only twenty two women had ever heard of breast ultrasonography, yet they were more likely to be aware of mammography compared to those who lack knowledge on breast ultrasonography. There were no significant associations between mammography awareness and worry about breast cancer, having had a physical examination in the preceding year before interview or reports of difficulty getting a physical examination.

**Table 3:** Relationship between awareness of mammography and variables related to attitudes towards mammography and health service utilization

**Variable**	**Aware of mammography n (%)**	**Total**	**P value**
**Ever worry about breast cancer**			
Yes	8(6.1)	131	0.582
No	34(4.9)	687	
**Chances of having breast cancer?**			<0.001
Likely	8(25.0)	32	
Unlikely/God Forbid	33(4.4)	753	
Don’t know	1(3.0)	33	
**Heard or seen anything about breast cancer prevention in the community?**			
Yes	12(17.9)	67	<0.001
No	30(4.0)	751	
**Aware of breast ultrasonography?**			
Yes	14(63.6)	22	<0.001*
No	28(3.5)	796	
**Had physical examination last 12 months**			
Had examination connected to a health problem	22(5.1)	429	0.979
Had examination but not connected to a health problem	2(5.9)	34	
Had no examination	18(5.1)	355	
**Difficulty getting a physical examination?**			
Yes	12(4.0)	302	0.138
No	29(6.5)	447	
**Ever had breast examined by a doctor or nurse for lump?**			
Yes	20(11.4)	176	<0.001
No	22(3.4)	642	
**Know that frequent breast examination can detect breast cancer early?**			
Yes	38(5.9)	641	0.111
No	0(0.0)	41	
Don’t know	4(2.9)	136	
**Practice BSE?**			
Yes	29(6.9)	421	0.019
No	13(3.3)	397	

BSE- Breast self -examination.

Using a multiple logistic regression model, awareness of mammography was regressed on variables which were significant at 5% level on chi square tests. The odds ratios and confidence intervals from the regression analysis are shown in Table 4. Those with primary or secondary levels of education were about three times less likely than those with tertiary level of education to be aware of mammography (95% CI, 0.12 – 0.73) while those with 1–4 children were about nine times more likely to be aware of mammography than those with 5 or more children (95% CI, 1.04 – 73.65). Exposure to any information about breast cancer prevention had an odds ratio of about 3.4 of being aware of mammography compared to those without such exposure (95% CI, 1.39 – 8.36). Women who had previous breast examination by a doctor or nurse were also more likely to be aware of mammography than those who never had previous breast examination (OR = 2.34, 95% CI, 1.10 – 4.96). An odds ratio of about 14.9 (95% CI, 1.10 – 4.96) was obtained for knowledge of any of the risk factors for breast cancer.

**Table 4:** Odds ratios and 95% Confidence intervals for regression of mammography awareness on selected variables

**Variable**	**Odds ratio**	**95% CI OR**	**P value**
Age(years)			
Less than 40	3.01	0.24 – 38.34	0.396
40-59	1.78	0.14 – 22.07	0.653
60+ (ref)*	1		
Education			
None	0.55	0.09 – 3.32	0.518
Primary or Secondary	0.30	0.12 – 0.73	0.008*
Tertiary(ref)	1		
Parity			
None	3.97	0.42 – 37.44	0.228
1-4	8.77	1.04 – 73.65	0.046*
5 + (ref)	1		
Had discussions about breast cancer with other women			
Yes	1.32	0.61 – 2.85	0.480
No(ref)	1		
Heard or seen anything about breast cancer prevention in community			
Yes	3.41	1.39 – 8.36	0.007*
No(ref)	1		
Self perceived Chances of having breast cancer			
Likely	8.34	0.76 – 91.69	0.083
Unlikely/God Forbid	0.89	0.10 – 8.05	0.914
Don’t know(ref)	1		
Know one or more symptoms of breast cancer			
Yes	1.27	0.56 -2.86	0.566
No(ref)	1		
Know one or more risk factors for breast cancer			
Yes	14.93	1.90 -111.11	0.010*
No(ref)	1		
Had Clinical breast exam			
Yes	2.34	1.10 – 4.96	0.027*
No(ref)	1		
Practiced self breast examination			
Yes	0.98	0.43 -2.22	0.953
No(ref)	1		
Source of information about breast cancer			
Newspaper/magazine	0.81	0.16 – 4.11	0.802
Radio/television	0.31	0.06 – 1.55	0.151
Friends/Colleagues	1.28	0.28 – 5.78	0.747
Hospital	0.44	0.08 – 2.46	0.351
Others	2.03	0.48 – 8.62	0.338
Never heard about cancer recently(ref)	1		

*ref indicates reference category.

## Discussion

Our study has found a low level of awareness of mammography and important socio-demographic and health service utilization variables predicting this level of awareness. The proportion of women who had ever heard of mammography was about 5% in this study. This figure contrasts with much higher proportions reported from other authors in Nigeria likely due to variability in the characteristics of the study population. Osime *et al. *[18] found a prevalence of mammography awareness of about 35% among civil servants while Akinola *et al. *[9] reported an awareness level of 40.5% among a hospital sample. The higher proportions from these studies are most likely a reflection of the high level of education of the samples studied where at least 60% of the samples had tertiary level of education compared to 32% from this study and a greater proportion of skilled workers [9]. Though our study found a prevalence of mammography awareness of about 11% among women with tertiary level of education, this group of women still has the highest frequency, thirty (71.4%) out of 42 women who have ever heard about mammography. This is quite close to reports by Akinola *et al.*[9] where 77.1% of women who have heard about mammography had tertiary level of education.

This study demonstrated very poor knowledge among this hospital population which is disappointing given the exposure to health education session and also contact with health workers such as doctors and nurses. A low level of skills in conducting health education by health staff and poor attitude of health workers which has been previously documented in Nigeria may contribute to this [10]. In fact, Onwere *et al.*[19] found that only 1% of women attending antenatal care had their breasts examined by doctors while nurses had examined 2% of the antenatal clinic attendees. Bello *et al.*[10] found that only 3.3% of medical practitioners referred patients for yearly mammograms. The failure of health staff to educate patients on important issues such as cancer screening greatly reduces the opportunity which hospital attendees have to gain information on health related issues.

This study found that none of the women had ever had a mammography done. Very low proportions of women from other studies reported they had mammograms [5,6,9]. The likelihood of participants having had mammograms is probably influenced by inclusion of female health workers [5,6]. Ibrahim & Odusanya [5] and Odusanya & Tayo [11] found that 8% of female health workers and nurses respectively ever had a mammogram while 3.1% of female health workers aged 40 years and above in another study reported they had mammogram [6]. In our study, the occupation of the respondents was however not considered.

The findings from association between mammography awareness and variables revealed that education was strongly significantly associated with mammography awareness. Similar finding was also reported by Akinola [9] and Lee *et al.*[12]. Educated women are more likely to benefit from most messages concerning breast cancer knowledge and methods of prevention and thus more likely to learn about mammography. Older women were less likely to be aware of mammography even though this was not significant on logistic regression analysis. The lack of association following logistic regression analysis may be due to a strong relationship between age and level of education. The low level of awareness among the older women is a reflection of the low literacy rate (less than 30%) among older female adults in the Nigerian society [20] and the poor level of enlightenment for the mammography screening procedure. Record show that the women most likely to benefit from mammography are those aged 50 to 69 years because of high prevalence of low breast density [21], however our study revealed that this age group demonstrated a very low level of mammography awareness. Women with less than five children were more likely to be aware of mammography compared to those with higher parity though the confidence interval was wide hence the low precision of the estimate. This finding may be a reflection of a group of women with better health knowledge and practices including child spacing and family planning.

Those who had been exposed to some information on prevention in the community were more likely to be aware of mammography. This is an interesting finding which underscores the need for more community based interventions on breast cancer awareness. Women who had clinical breast examination in the past were also more likely to be aware of mammography screening procedure. The utilization of other breast cancer screening services can serve as a veritable means of obtaining information concerning screening methods such as mammography. The fact that women with poor access to physician care are less likely to undergo mammography has been reported by Schueler *et al. *[16]. Clinicians need to be educated on the need to give information to women on the most current investigative or screening methods as these patients may never get such opportunities in other settings apart from hospitals. The possibility of improving mammography screening among hospital inpatients has also been described elsewhere [22].

Family history of cancer was not significantly associated with awareness of mammography. Similar results were found by Akinola *et al. *[9] and West *et al. *[14]. One would have expected a higher level of anxiety among women with family history of breast cancer which should translate to a higher level of mammography awareness. However fear of having a positive screening test could make utilization of breast cancer screening services low [23].

One of the limitations of this study included the fact that it is hospital based and may not truly reflect the level of awareness among the general population. In addition, some of the confidence intervals were wide indicating moderate degree of uncertainty in the estimates. This is an indication of the relatively low proportion of about 5% who had the outcome.

## Conclusion

This study has provided some information about factors influencing mammography awareness in a developing country. Important opportunities for interventions have been identified based on these factors. Clinicians and other health workers have an important role to play in improving the level of awareness concerning mammography especially among hospital attendees. As this study has found no significant difference in the level of mammography awareness between those with family history of breast cancer and other women, attention should be given to the former by establishing a genetic counseling unit. Interventions promoting awareness of this screening procedure should give particular attention to the illiterate and older women while opportunities are given to public health educationists and community health extension workers to reach the public.

## Competing interests

The authors declare that they have no competing interests.

## Authors’ contributions

MOO and BOA were involved in the conceptualization of the study. MOO, IOA and AOO participated in the design of the methodology including the data collection instruments. AAS, OAM and TSA helped in the collection of the data and preparation of the manuscript. BOA performed the data analysis and wrote the results section. FA, IO and CU collected data and helped in the preparation of the manuscript. OSB, EU and TOS did the literature search and wrote the first draft of the manuscript. AOO and OIO reviewed the manuscript for intellectual contributions. All authors read and approved the manuscript.

## Pre-publication history

The pre-publication history for this paper can be accessed here:

http://www.biomedcentral.com/1471-2458/13/40/prepub
